# Executive functions and emotional self-regulation in children and adolescents

**DOI:** 10.21500/20112084.7631

**Published:** 2025-12-04

**Authors:** Carmen Graciela Zambrano-Villalba, Iván Leonardo Pincay-Aguilar, Saulo Sánchez-Zambrano

**Affiliations:** 1 Universidad Estatal de Milagro. Milagro, Guayas, Ecuador. Universidad Estatal de Milagro Universidad Estatal de Milagro Milagro Guayas Ecuador; 2 Universidad de Especialidades Espíritu Santo. Guayaquil, Ecuador. Universidad de Especialidades Espíritu Santo Universidad de Especialidades Espíritu Santo Guayaquil Ecuador

**Keywords:** Neurodevelopment, executive functions, learning, children, adolescents, emotional self-regulation, Neurodesarrollo, funciones ejecutivas, aprendizaje, niños, adolescentes, autorregulación emocional

## Abstract

The objective of the study was to analyze the relationship between executive functions and emotional self-regulation in the learning process of children and adolescents. The method used was quantitative, non-experimental, cross-sectional and correlational, with 531 participants. Tests validated in the Ecuadorian contact were used for cognitive functions and emotional self- regulation. The results showed that executive functions have a medium (5 6), high (8) and very high (9-10) development, in the age groups analyzed, significant differences stand out in phonological fluency (51%) more in women than in men (31%) as well as semantic fluency 49% of men reach medium- high levels, compared to 34% of women, performance associated with the capacity of association, organization of information in semantic categories added to working memory and cognitive flexibility. It is concluded that there were differential patterns with those participants who were associated with the diagnosis of ADHD and a very significant relationship (.756**) between executive functions and emotional self-regulation in both groups.

## Resumen.

El estudio analizó la relación entre funciones ejecutivas (FE) y la autorregulación emocional (ARE) y su implicación en el proceso de aprendizaje en niños y adolescentes. Se empleó un diseño cuantitativo no experimental de corte transversal, descriptivo y correlacional, la muestra estuvo compuesta por 531 participantes ecuatorianos, a quienes se le administraron pruebas validadas localmente para funciones cognitivas y autorregulación emocional. Los resultados descriptivos, revelaron un nivel de desarrollo de las funciones ejecutivas medio (5-6), alto (8) y muy alto (9-10), en los grupos etarios analizados, se observaron diferencias significativas en fluidez fonológica (51% más en mujeres que en hombres 31 %) y la fluidez semántica (49 % de los varones vs 34 % mujeres en niveles medios altos), en el análisis mediante Modelos de Ecuaciones Estructurales se reveló una relación predictiva significativa de las Funciones Ejecutivas sobre la Autorregulación Emocional (p=.68, p < .001). Específicamente, se identificó que la Memoria de Trabajo y la Flexibilidad Cognitiva son los componentes de las FE que más contribuyen a la varianza de la ARE. Además, se encontró que el diagnóstico asociado de TDAH modera la relación entre FE y ARE, mostrando una asociación significativamente más débil en este subgrupo. Se confirmó una correlación positiva y altamente significativa en la muestra total (r = .756, p < .01). Estos hallazgos reafirman la interdependencia de los dominios cognitivo y emocional y subrayan la necesidad de programas de intervención integrales y diferenciados según el perfil neurocognitivo en el contexto educativo.

## 1. Introduction

In recent decades, there has been a growing interest in assessing Executive Functions (EF) in children and adolescents due to their central role in cognitive and emotional development within the learning process. EF constitute a set of crucial abilities, such as verbal fluency, working memory, planning, inhibition, and cognitive flexibility, which enable students to organize information in a coordinated manner and regulate their behavior to cope with daily situations (Kim et al., 2020). Adequate executive functioning is essential, as its effectiveness directly ensu res good academic performance (Kim et al., 2020; Montoya-Arenas et al., 2018).

The objective of this research was to analyze neurodevelopment by determining the relationship between executive functions and emotional self-regulation in the learning process of children and adolescents. This analysis aimed to characterize the neuro development level of EF and, based on parent perception establish the correlation between EF levels and learning condition, EF are crucial as Dorman et al. (2022) define them as the human brain's capacity to transform thoughts into actions in an organized, flexi ble, and effective manner, leading to the individual's adaptation to circumstances. Thus, EF constitute the general system that regulates, controls, directs, and evaluates actions, allowing or inhibiting behaviors as necessary.

Learning in childhood depends on various neurocognitive factors that influence children's ability to process and acquire knowledge. Within these, executive functions play a crucial role in facilitating skills such as working memory, inhibitory control and cognitive flexibility, essential for academic success (Bernal-Ruiz & Cerda, 2024). These functions allow planning, organizing, making decisions and adapting to the demands of the educational environment, highlighting its relevance in the school environment and its relationship with academic performance.

Luria (1979) highlighted the role of the frontal lobe in behavioral control, laying the foundation for understanding how executive functions regulate behavior and emotions. Later, Lezak (1982) defined these functions as a set of skills necessary to plan and achieve objectives, which is essential in the learning process. Subsequent research has linked the development of executive functions to emotional self-regulation (Pas et al., 2021; Koay & Meter, 2023), highlighting its influence on attention (Abou Sleiman & Kechichian Khanji, 2021) and on children's ability to face academic challenges effectively (Saadi, 2025).

Cognitive development is related to executive functions, which occurs due to neurobiological activity stimulated by the family and school social context, which allows the development of self-control and self-regulation of learning (Sankalaite et al., 2021). As children grow physically and become more involved in learning activities, skills such as inhibition, working memory, cognitive flexibility, motor control, planning, and organization gradually improve. From the earliest years, children acquire basic knowledge necessary for the continuity of learning throughout life (Wang et al., 2023; Arán-Filippetti & Krumm, 2013).

Therefore, cognitive development and subsequent performance during the formative years depend on the information that children absorb (Piaget, 1937) and the maturation and functioning of executive functions, as well as the influence of social and emotional interactions (Zhengxian et al., 2024), since it is essential to achieve good development of executive functions for the acquisition of emotional self-regulation, a situation that is evidenced behaviorally in interrelationships with peers and in the process of learning. This comprehensive approach highlights the need to consider EF as a bridge between cognitive and socio-emotional development (Cui et al., 2022).

Furthermore, recent studies have confirmed that environmental factors, such as the family and school context, play a significant role in the development of executive functions (Lynch et al., 2024). They point out that positive interactions with caregivers, along with a structured environment, promote the development of skills such as planning and inhibitory control. Likewise, exposure to pedagogical practices based on play and problem solving stimulates the development of these functions, especially in the early school stages. In this direction, Allee et al, (2023) stated that children ex- posed to a play-based pedagogical approach showed better EF health and greater academic gains compared to those who received a more conventional pedagogical approach.

From a neuropsychological perspective, Jones and Graff-Radford, (2021) highlight that executive function encompasses working memory, cognitive flexibility and inhibition and that its regulation is not limited to the frontal lobe, but depends on distributed brain networks. They also describe the key role of the prefrontal cortex in cognitive, social, emotional and motivational functions, pointing out how different areas participate in planning, attention, inhibition and emotional regulation. The authors highlight the complexity of evaluating executive functions from a multidimensional approach to capture the interaction between cognitive, emotional and behavioral components.

Cabrera et al, (2024) stated that a neuroeducational approach in the classroom, guided by trained teachers, transforms learning and overcomes the limitations of the traditional educational system, because the adequate development of executive processes allows children to recognize and mentally represent various problematic situations posed by the teacher from this perspective, as well as the advances in cognitive neuroscience. In educational research, it is becoming increasingly clear that stimulating executive functions can have a significant impact on student learning. Therefore, the teacher must take into account these specific circumstances of the students to reflect and offer the content that must be addressed in order to better connect with their students and promote meaningful learning. (Sankalaite et al., 2021).

At the educational level, strengthening executive functions has been identified as a key fac tor to reduce learning gaps and promote equity. Tools of the Mind was a pedagogical program designed to improve self-control and working memory, it has shown positive results in children from disadvantaged backgrounds (Goble et al., 2021). These findings highlight the relevance of designing educational interventions that consider executive functions as a central axis for the comprehensive development of students.

Vasquez & Marino, (2021) state that there is no doubt that executive functions play a crucial role in learning. Cognitive skills related to executive function, such as self-regulation, planning, decision making, and working memory, are essential for the efficient acquisition, processing, and retention of new information. They also promo te attention and concentration, thus improving the ability to process information in a deep and meaningful way. Likewise, organizing and planning executive function supports that the ability to plan and organize time and resources is essential for the effective performance of educational research and academic tasks.

## 2. Methodology

The non-experimental quantitative design was used, with a cross-sectional descriptive-correlational scope. The population was made up of a total of 2,139 students from the provinces of Bolívar, Pichincha, and Guayas, Ecuador, of which 519 were from municipal educational units and 1,120 from public educational units, from rural and urban areas, and 500 children from centers specialized in neurodevelopmental problems. The technique for sample selection was non-probabilistic for convenience. The inclusion criterion was established to be two groups of school participants without previous diagnoses of neurological disorders and another group with a confirmed diagnosis of ADHD according to DSM-5 criteria (American Psychiatric Association, 2013) from different age groups to carry out the comparisons of 6-12 years and 13 to 17 years of basic general education and high school, prior informed consent for participation. As an exclusion criterion, students who did not send said consent, students with serious communication difficulties, were considered. or interaction that make the evaluation process difficult, leaving a total of 531 participants, of which 331 were without neurodevelopmental problems and 200 diagnosed with ADHD, of which 170 were men and 361 women.

For data collection, the Neuropsychological Assessment of Executive Functions in Children (ENFEN) questionnaire, created by Portellano et al. (2009), was used. This tool assesses phonological fluency (F1) and semantic fluency (F2), fluency in the gray path (S1) and the chroma- tic path (S2), fluency in the gray path (A), and resistance to interference (I). The results are reported using decatype levels: very high (9-10) which is equivalent to excellent, high (7-8) equivalent to above-average performance compared to peers, medium (5-6) equivalent to nor mal expected performance, decatype (3 4) below average, and (1-2) significant deficit. This questionnaire includes scales tailored for specific ages (6-12 years) and shows high internal consistency (Cronbach's alpha = 0.8) according to the authors. In the Ecuadorian context, a Cronbach's alpha of 0.80 was obtained, which provides reliable data for the continuity of the research.

The Behavioral Assessment of Executive Functions questionnaire (BRIEF®-2) was used, applied to the representatives; This instrument has a total of 63 items with 3 response options 1 (has never been a problem)2 (sometimes)3 (frequently), divided into 9 subscales, inhibition (inh), self-monitoring (smi) which belongs to the behavioral domain; flexibility (flx), emotional control (Cem) that belongs to the emotional domain; initiative (ini), working memory (Mtr), planning and organization (Pla), task supervision (Sta), organization of materials (Org), which belongs to the cognitive do- main. The questionnaire adapted to the Spanish population has a Cronbach's alpha (a = .86) (García et al., 2014; Gioia, et al., 2017). In the present investigation, a reliability coefficient of (a = .98) was obtained.

The “Emotional Self-Regulation Questionnaire” (ERQP) by Gross & Thompson (2003) and adapted in Peru by Gargurevich and Matos (2010) was used, which has a to tal of 10 items with a two-factor structure: cognitive reappraisal 6 items and suppression 4 items. Scores range from 1 (strongly agree) to 7 (strongly disagree). Regarding Cronbach's alpha obtained in Peru, it was found (a = .72) for the cognitive reappraisal scale and (a = .74) for the suppression scale. As there are no studies at the Ecuadorian level, Cronbach's alpha was obtained, resulting in (a = .73) in both components.

For statistical analysis, the SPSS program was used, which allowed for the evaluation of frequencies, correlations, means, standard deviations, as well as bivariate inferential analyses, Pearson's test, and the correlation coefficient (r). A significance criterion of p < 0.05 was used. Subsequently, the means were compared simultaneously by sex, and the structural equation model was incorporated to establish causal relationships between the variables, confirming the internal consistency that allowed us to demonstrate that executive functions predict or cause changes in academic performance and their mediation or moderation of learning in schoolchildren, determining the changes that may occur in children diagnosed with ADHD.

Before starting data collection, permission was obtained from the authorities of the participating institutions, information was pro- vided on the confidentiality of the information provided, in order to safeguard personal integrity, warning that said data will be used solely and exclusively for research purposes, in addition to the informed consent of pa- rents or legal representatives, guaranteeing compliance with the ethical principles established in the Declaration of Helsinki. Special care was taken to ensure the confidentiality and anonymity of the data obtained, in order to protect the rights and well-being of the children participating in the research.

## 3. Results

**Table 1:** Results of the neuropsychological evaluation of Executive Functions (ENFEN)

Subcomponent		Decatype		Percentages		
	3	4	5-6	7	8	9-10
F1	^-^	^-^	23	13	20	43
F2	^-^	3	30	13	13	40
S1	^-^	^-^	13	13	33	40
S2	^-^	^-^	34	30	13	24
A	10	17	27	17	27	3
I	^-^	^-^	27	17	7	50

According to the results, a good level of development of executive functions is observed that vary between decatype 9-10 (very high), 8 (high) and 5-6 (medium), thus 43 % of the participants have a very high level of verbal fluency, while 20 % obtain a high verbal fluency, 30 % obtain a medium level, likewise in semantic fluency and gray paths, 40% have a very high level while in color paths they are at the medium level. In rings, the distribution is at a medium and high level with 27 %. Respectively, but it must be noted that, although it is not very representative, 10 % are in decatype 3 and 17 % in decatype 4, which represent very low levels. In the interference subtest, 50 % are positioned in decatype 10, considered very high, while 27% are at the medium level.

**Table 2:** Results of the neuropsychological evaluation of Executive Functions in relation to gender

Subc	3	4	5-6	7	8	9-10
	H-M	H-M	H-M	H-M	H-M	H-M
F1	1-0	8-11	13-16	48-21	31-33	31-51
F2	^-^	6-8	9-10	49-34	33-27	3-21
S1	^-^	8-14	23-17	19-42	41-21	9-6
S2	^-^	12-8	16-16	29-38	38-27	5-11
A	^-^	12-17	23-18	31-39	25-21	9-5
I	^-^	12-11	30-35	32-38	17-19	8-7

The Phonological Fluency test denotes that 51% of women are at higher levels than men, reaching 31%, indicating that female participants have superior performance in skill in tasks related to structured verbal production under cognitive pressure. In terms of semantic fluency, 49 % of men reach medium-high levels, compared to 34% of women. Here it is shown that children have better performance in this category, which could be associated with a greater capacity for association, organization of information in semantic categories, in addition to working memory and cognitive flexibility. Differential patterns were found with those participants who were associated with the diagnosis of ADHD, a key point to establish neuropsychic-pedagogical intervention strategies that develop compensatory strategies that favor their perfor mance in language-related tasks.

The gray trail test was found distributed in the high averages and high averages for both women (42%) and men (41%) together, it could indicate a better capacity for sustained attention, cognitive flexibility, attentional alternation and processing speed in both girls and boys. skills that are fundamental in school and social life. The Ring test shows that a high average distribution of 39% in women as opposed to 31% in men, this indicates that there is greater per formance in the skills related to problem solving, sequential organization, and effective planning and organization. In the interference test, medium, high and very high levels were reached in women than in men, indicating similar perfor mance in both genders, although women show a slight advantage at the very high level and men at the medium-high level. This equivalence suggests that both genders have a comparable percentage of individuals with outstanding abilities to inhibit automatic responses and handle tasks that require cognitive flexibility. This ability to inhibit impulsive responses and maintain attention on relevant stimuli is usually affected in children with ADHD. However, the results reflected that both genders have a comparable percentage at very high and high levels, which suggests that, when appropriate strategies are developed, both girls and boys can achieve effective control of impulsive responses.

**Table 3:** Results of the behavioral evaluation of executive functions from the perception of parents

Subscale	Responses		Subscale	Responses	
Inh	70	30	Mtr	87	13
Smi	80	20	Pla	77	23
Flex	75	25	Sta	70	30
Cem	70	30	Org	75	25
Ini	85	15			

Regarding the development of executive functions from the perception of parents, the results demonstrate normal indicators in relation to age and gender; In the behavioral domain, the majority of legal representatives mention that their children do not have learning problems (70%); However, a small group of representatives identified that their children face certain difficulties in this area (30%). They require accompaniment to carry out their schoolwork, which could indicate a significant level of dependency when studying, only 20% carry out their activities with self-supervision, which corresponds to adolescents, while 80% of minors require accompaniment. In the cognitive domain, it was found that 75% processes and organizes information flexibly, most of the participants, 25% were accompanied by indicators of distractibility, lack of interest and behavioral difficulties associated with problems in the emotional domain, 30% imperceptible to others in their environment but which significantly affects academic activities.

The results show a profile of impairment in the global index of mild executive function associated with the ability to control behavior, flexibility, emotional control, initiative, working memory, planning, self-supervision and task supervision where a minority percentage require supervision of both the normal group and those diagnosed with ADHD, which clearly indicates problems in cognitive regulation, autonomy, independence and self-confidence, frequent distraction and forgetting what they have learned, presence of impulsive behaviors, associated with socio-familial, socio-emotional and socio-school factors. It is highlighted that despite this minimal impairment, they do not present low indicators for solving problems or adapting to new tasks, which suggests a good base of cognitive and learning skills.

**Table 4:** Results of the evaluation of emotional self-regulation in relation to age and gender

Variable	M	DS	t	p	IC		Age Gender	
Cognitive		19.47	5.17	-2319	,023*	(-4.45, -0.35)	-0.178	1.004
Reassessment								
Emotional		18.42	.79	-2.157	,033*	(-4.78, -0.20)	-0.225	-1.824
Suppression								

In relation to cognitive reappraisal, participants showed significantly higher levels than in emotional suppression. Analysis using the t test for independent samples corroborated the statistical significance of this difference (t(df) = -2.319, p = .023), with a mean difference of -2.40 (95% CI [-4.45, -0.35]). These variations could be attributed to specific contextual factors, including differences in educational environments, available psycho-pedagogical resources, and sociocultural characteristics specific to each institutional environment.

**Table 5:** Correlational and structural equation mode- ling results between FE and ARE

Predictive Path (Pathway)	P **(Standardized** **Coeff.)**	Critical Ratio (t)	p-value
Executive Function righters Emotional Self-Regulation	.68$	$13.60$	$<.001$
Working Memory $\rightarrow$ Emotional Self- Regulation	$.42$	$6.00$	$<.001$
Cognitive Flexibility $\rightarrow$ Emotional Self- Regulation	$.30$	$5.00$	$<.001$
Emotional Self-Regulation	Pearson Correlation Sig	1	,756** ,001
Executive Functions	Pearson Correlation Sig	,756** ,001	1

The Structural Equation Modeling (SEM) analysis revealed that Executive Functions (EF) are a significant and robust predictor of Emotional Self-Regulation (ESR) across the total sample, as indicated by the highly significant standardized coefficient ($\ beta = .68, t = 13.60, p < .001$). Upon examining the specific components of EF, a differential contribution of each factor to the model was identified. Specifically, Working Memory proved to be the strongest predictor of ESR ($\beta = .42, t = 6.00, p < .001$), suggesting that the capacity to actively maintain and manipulate information is fundamental for emotional modulation. Secondly, Cognitive Flexibility also exhibited a significant contribution ($\beta = .30, t = 5.00, p < .001$), con- firming that the ability to shift perspectives and mental strategies is essential for adapting to changing emotional demands. These results establish a clear explanatory model of the neurocognitive and emotional interdependence in children and adolescents.

It was determined according to the results that there is a very significant positive correlation between executive functions and the process of emotional self-regulation in students within the learning process (r=0.756**, p < 0.001). These results suggest a trend towards a differential effect within emotional self-regulation on cognitive reappraisal and emotional suppression that affects the execution of executive functions depending on age and gender, a situation that should be explored in future research.

## 4. Discussion

The findings of this study highlighted the importance of executive functions (EF) in relation to emotional self-regulation within the learning process in children and adolescents. Executive functions were identified as significant predictors of the learning process, which coincides with previous research that indicates that skills such as working memory, inhibitory control and cognitive flexibility have a direct impact on school perfor mance (Cheung & Chan, 2022). Likewise, it is evident that these skills can be developed through structured and appropriate pedagogical practices (Saadi, 2025).

Recent studies (Toh & Yang, 2022) highlight that cognitive reappraisal, an adaptive strategy for emotional self-regulation, is significantly associated with better executive functioning in daily life. This suggests that individual differences in executive abilities may mediate the effectiveness of emotional regulation strategies. Specifically, Executive Functions (EF) such as inhibitory control (which allows the suppression of maladaptive responses), working memory (which facilitates the manipulation of cognitive strategies), and cognitive flexibility are fundamental for regulation. The statistically significant correlation found between these dimensions reaffirms their positive association (Toh & Yang, 2022). Furthermore, cognitive flexibility has been identified as being more strongly associated with emotional regulation in contexts where adaptability is essential (Wang et al., 2024).

Research such as that of (Sousa et al., 2023; Medrano et al., 2023) has shown that these variables can be clearly related in clinical populations or in longitudinal studies. This points to the need to consider additional factors, such as the level of academic stress, sociodemographic characteristics, and specific self-regulation strategies used by children and adolescents. In the study carried out by (Kraft & Grant, 2021), it is mentioned that the factors that influence executive functions in people with social anxiety disorder include greater difficulties in emotional regulation and worse executive function skills compared to those without this disorder. Deficits in emotional control were associated with the inhibition process and the age of onset of the disease. Likewise, this becomes a social influence factor that causes variations in the emotional self-regulation of children and adolescents, which affects aca- demic performance and the learning process.

Cognitive reappraisal and emotional suppression are evident in the process of emotional self-regulation, and the development of emotional strategies is important in educational contexts (Smith and Green, 2020). In addition, they are considered key strategies in the process of emotional self-regulation, playing a fundamental role in educational contexts (Andrés et al., 2020). However, the effectiveness of cognitive reappraisal may be limited by individual differences in its application and sustainability over time. As has been suggested in recent studies, although this strategy momentarily improves emotional states in controlled environments, its im- pact on emotional regulation in future situations is not guaranteed (Wang & Yin, 2023).

In relation to cognitive reappraisal, they showed significantly higher scores in relation to age and gender, which could be interpreted as a greater ability to interpret emotional situations in a positive way. This finding suggests that participants tend to employ more adaptive emotion regulation strategies. In this sense, the greater availability of emotional support and educational programs could facilitate the acquisition of cognitive reappraisal skills, promoting a healthier emotional response to stressful situations. These results coinci de with the findings of (Mohan et al., 2024).

On the other hand, emotional suppression, although generally considered a less adaptive strategy, also showed significant differences between the groups, indicating a tendency to suppress their emotions in difficult situations. This pattern may reflect differences in the sociocultural and psychological environments that affect adolescents, such as external pressures and limited resources in some educational contexts (Liu et al., 2024).

## 5. Conclussions

The results of this study robustly confirm the critical interdependence between Executive Functions (EF) and Emotional Self-Regulation (ESR) in children and adolescents, evidenced by a positive and highly significant correlation (r = .756). The analysis using Structural Equation Modeling (SEM) goes beyond mere association, establishing EF as a significant predictor of ESR. This finding validates theoretical frameworks positing a shared neuro- cognitive basis for behavioral control and emotional modulation, suggesting that the efficacy of the learning process is intrinsically dependent on this synergy of skills.

The structural model allowed for the identification that Working Memory and Cognitive Flexibility are the EF components that contribute most significantly to the variance in Emotional Self-Regulation, highlighting their essential role in adopting adaptive strategies. Based on this evidence, there is a compelling need to design integral psychoeducational and neuropsychological interventions. These interventions must focus on simultaneously strengthening the capacity to manipulate in- formation (Working Memory) and the ability to shift perspective (Cognitive Flexibility), aiming to improve emotional competencies and, consequently, academic performance within the school setting.

Although the cross-sectional design allowed for establishing a strong predictive relationship, the inability to deter mine long-term causality is acknowledged as a limitation. Therefore, future research should adopt longitudinal and mixed approaches that allow for monitoring the evolution of these relations- hips and more precisely establishing the mechanisms of mediation or moderation, particularly in populations with atypical developmental profiles, such as TDAH. It is essential to build more sophisticated theoretical models that capture the complexity of neurodevelopment and guide clinical and educational practice toward more differentia- ted and effective support models.

## 6. Remark

This article is part of the work carried out in 2024 within the research module I and II neuropsychology of learning of the graduate faculty of the State University of Milagro. Guayas Ecuador.
